# Qresp, a tool for curating, discovering and exploring reproducible scientific papers

**DOI:** 10.1038/sdata.2019.2

**Published:** 2019-01-29

**Authors:** Marco Govoni, Milson Munakami, Aditya Tanikanti, Jonathan H. Skone, Hakizumwami B. Runesha, Federico Giberti, Juan de Pablo, Giulia Galli

**Affiliations:** 1 Institute for Molecular Engineering and Materials Science Division, Argonne National Laboratory, Lemont, IL, 60439, USA; 2 Institute for Molecular Engineering, University of Chicago, Chicago, IL, 60637, USA; 3 Research Computing Center, University of Chicago, Chicago, IL, 60637, USA; 4 Department of Chemistry, University of Chicago, Chicago, IL, 60637, USA

**Keywords:** Materials science, Research data, Publishing

## Abstract

We propose a strategy and present a simple tool to facilitate scientific data reproducibility by making available, in a distributed manner, all data and procedures presented in scientific papers, together with metadata to render them searchable and discoverable. In particular, we describe a graphical user interface (GUI), Qresp, to curate papers (i.e. generate metadata) and to explore curated papers and automatically access the data presented in scientific publications.

## Introduction

The reproducibility of experimental and computational results presented in scientific papers is an important and critical part of the overall research process. Yet the data discussed in most scientific papers are not made available to the community, and the procedures followed to generate the data are often not articulated step by step, or in any detail^1–3^. Sometimes only small datasets or partial procedures are described in the supplemental information (SI), in no particularly organized manner and with no metadata available.

There are growing concerns in the scientific community about data that cannot be reproduced, affecting the validity and accuracy of scientific findings, as described in many recent papers and comments in leading scientific journals^4–9^ and highlighted by several conference organizers. Some conference proceedings are now requiring information about data reproducibility; for example, the international Supercomputing conference series that typically attracts more than 10,000 people introduced the reproducibility initiative in the 2016 technical papers program (https://sc18.supercomputing.org/submit/sc-reproducibility-initiative/). In addition, many research groups often encounter difficulties in finding or recreating their own data^10^ and the peer-review process may be difficult, since often times procedures are not readily accessible and necessary data are either missing or reported utilizing digital formats which are not machine-readable^11^. Equally important is the difficulty in analyzing and using data produced by other researchers, effectively harming (or at least slowing down) data mining and statistical learning activities, which are becoming an important part of many disciplines^12,13^ and require the availability of curated data.

Effective data management has the potential to increase the pace of scientific discovery and promote an efficient and effective use of funding and resources; hence several funding agencies are mandating the inclusion of data management strategies as an integral part of research planning (https://science.energy.gov/funding-opportunities/digital-data-management/), (https://www.nsf.gov/bfa/dias/policy/dmp.jsp). However, the lack of wide-spread social and technical infrastructure solutions that can assist scientists in gathering and organizing their research data, starting from those generated and used in published papers, is hindering the effectiveness of such initiatives.

In recent years, several data repositories have been built by different scientific communities, and the availability of data reported in published papers would greatly increase the scientific impact of these repositories, as discussed by several authors. For example, substantial progress in computational materials science and chemistry has been made in the last decade^14–18^ from the availability of data resources such as the Materials Project^19^, the Open Quantum Materials Database (OQMD)^20^, the NIST Materials Data Repository^21^ (https://materialsdata.nist.gov/), NREL MatDB National Renewable Energy Laboratory (http://materials.nrel.gov), MatNavi^22^ (http://mits.nims.go.jp/index_en.html), Nomad (https://nomad-repository.eu/), CMDNetwork (http://www.asminternational.org/web/cmdnetwork), Citrine Informatics (http://citrination.com), and the Materials Data Facility^23^ (https://www.materialsdatafacility.org). Similar efforts have been proposed in other disciplines^24,25^ and general scientific repositories such as Dataverse (http://dataverse.org), Figshare (https://figshare.com), and Zenodo (https://www.zenodo.org) have been developed.

The idea that we propose in this paper is to facilitate scientific data reproducibility by making available, in a distributed manner, all data and procedures presented in scientific papers, together with metadata to make them searchable and discoverable. To this end, we lay out a general strategy (i) to organize the data presented in scientific papers in a manner suitable to make them available to the public through web-based graphical user interfaces; (ii) to succinctly describe the experimental and/or computational procedures used to obtain the data, and (iii) to generate searchable metadata.

In order to enact the strategy outlined above, we devised a simple, graphical user interface (GUI), Qresp, to curate papers (i.e. generate metadata) and to explore curated papers and access the data presented in scientific publications. Using such GUI, the authors may easily make available to the community the data of each of their publications, together with options for fine-grained progressive exploration and searches of the metadata. For example, each figure and table will be reproducible; in addition, the curation GUI offers the option of creating workflows describing the specific procedures adopted to acquire or generate the data.

In the following, we describe the general open source software Qresp “*Curation and Exploration of Reproducible Scientific Papers*” (http://qresp.org/) and we give examples of curated papers and of the data which the community may have access to, by exploring curated papers. We then discuss general features of Qresp and future perspectives.

## Results

**Figure 1:**
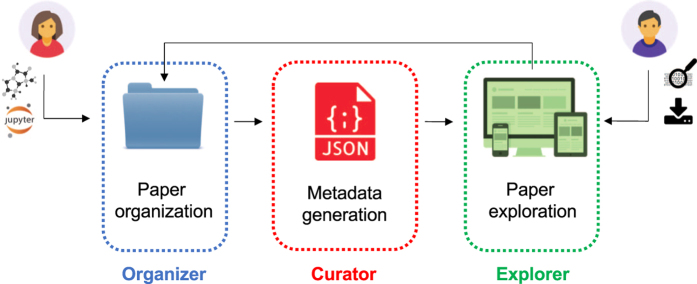
Summary of Qresp capabilities. Organization of data used and generated in a scientific paper (Organizer: see also Fig. 2); curation of data (Curator: see also Fig. 2) and exploration of papers (Explorer; see also Figs 3 and 4).

Qresp is an open-source web-based application that may be used to both curate and explore data presented in scientific papers or just explore curated scientific papers. The curation and exploration strategies are implemented in three steps, summarized in Fig. 1, and described in detail below. The strategy and the GUI are general and not restricted to papers of a specific domain.

### Paper organization

**Figure 2:**
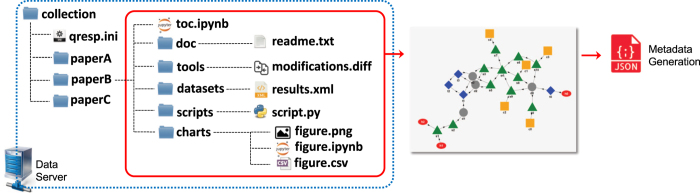
Proposed paper organization and curation. The left panel presents a proposed paper organization scheme (which is not mandatory for Qresp use; see text). The right panel shows an example of a simple workflow generated by curating a scientific paper. By hovering over a chart (yellow square), one will be presented with the specific table or figure, which was created with scripts (green triangles), acting on the specified datasets (gray circles). The datasets (gray circles) are the raw outputs created by an instrument – software or experiment – (blue diamond). The initial data, on which the investigation of the paper is based upon, are represented by the red external sources. The datasets are available for download when exploring the paper (see Fig. 3).

The first step in making data reproducible and sharable is data organization. The authors organize the data presented in a scientific paper in a manner of choice. An example is presented in Fig. 2: the data are organized as a collection of datasets (raw data acquired or generated for the paper, either as a result of a computation or collected by an instrument), charts (including images of figures and tables, notebooks used to create them and data displayed in the figure or table), scripts (codes not publicly available, used to manipulate datasets and generate the data files of charts), tools (publicly available software or facilities or instruments used to generate the data), and notebooks. The organizer feature of Qresp allows the authors to version and track changes of their data using Git (https://git-scm.com/).

There are no specific formats required for the files and for their organization, and the choice of the storage medium is made by the researcher, who may want to consider options such as self-hosted on-premise servers, different types of cloud storage or established centralized repositories. The long-term preservation of data depends on the terms and conditions of the storing strategy chosen by the authors. Given the generality and flexibility of the metadata file, Qresp can operate with a variety of platforms for data dissemination: links embedded in the metadata file point to the physical location where data are stored.

### Metadata generation

Once the data have been organized, e.g. as suggested above or in a manner of choice by the investigator, the GUI of Qresp guides the user in creating metadata from the data associated to a scientific paper. Qresp curator may automatically fill certain metadata fields and verify those inserted manually if an SSH connection to the server hosting the data is established. With this automation the users retain complete control over the curation process while minimizing their need to invest or acquire unnecessary technical skills. The metadata gathered during this curation step include data location, publication details and easily extendable user-defined attributes. The Qresp software also offers the option to generate, review and edit a data workflow that describes the procedure(s) followed to obtain the results discussed in the paper. An example is shown in Fig. 2. The metadata are generated using the JSON (JavaScript Object Notation, http://json.org/) syntax and the metadata file may be entered into a document-oriented database.

The choice of collecting metadata in a database, instead of the whole data, enables the authors to store their data in a location of choice, and to organize their data in the most appropriate way for their research. The overall strategy of Qresp is to provide a fully flexible tool, adaptable to diverse research needs, including multi-institutional and multi-disciplinary collaborations. Eventually established databases where the data and metadata of various publications are collected may be linked to each other and also linked to other databases of interest. For example, within the materials science community, one may plan to link the data and metadata of papers to the Materials Data Facility Materials Data Facility^23^ (https://www.materialsdatafacility.org), Nomad (https://nomad-repository.eu/) and/or other databases.

We emphasize the importance of generating workflows^3,26^, not only for tracing provenance of data and making them transparent to the community^27^, but also in order to explain in a detailed and compact way the scientific strategies used in the paper; these may then be used for training purposes for students or investigators interested in joining a specific project related to the paper, or for collaborations. In the future, the generation of the workflow can be automated by enabling Qresp to read the metadata produced by workflow management tools, e.g. Fireworks^28^ (https://materialsproject.github.io/fireworks/), AiiDA^29^ (http://www.aiida.net), Signac^30^ (https://glotzerlab.engin.umich.edu/signac/), Kepler (http://www.kepler-project.org), Galaxy^31^ (https://galaxyproject.org/). In addition, the specification used to encode the graph will follow portable and scalable schemes, e.g. the Common Workflow Language (https://www.commonwl.org/), to ease the re-execution or repurpose of curated workflows.

### Paper exploration

**Figure 3:**
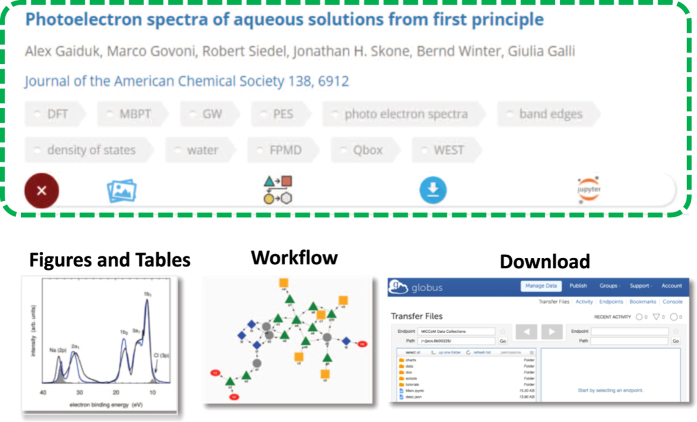
Example of how a paper, organized and curated as shown in Fig. 2, appears upon exploration using Qresp. The exploration GUI of Qresp provides a portal for the scientific community to access datasets, explore workflows and download the data published in scientific papers (see text).

**Figure 4:**
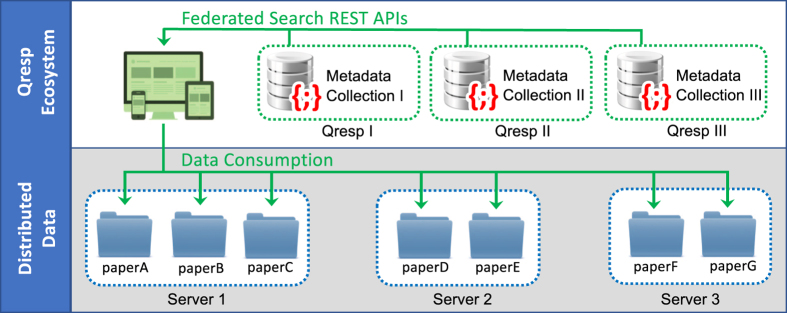
Scientific papers are searched and accessed utilizing a distributed model. Each search is dynamically translated into a query of the Qresp ecosystem, i.e. the collection of available Qresp instances. Each Qresp instance (node of the network) exposes REST APIs that facilitate the interaction with the underlying metadata collections. The resulting research data are automatically displayed by rendering the content stored in one or multiple servers.

The GUI of Qresp allows the user to search, browse, and access the curated scientific papers along with the datasets described in the paper. Within the exploration GUI, every entry is linked to a publication and is comprised of charts (figures and tables), workflows, notebooks, and datasets (see Fig. 3). Users may discover curated papers using a federated search (see Fig. 4), view charts, notebooks, and workflows on a per publication basis, and download the data organized as outlined above. Charts can be downloaded; the user has access to the file that contains the digital data of the picture (e.g. in CSV format), the notebook (e.g. Jupyter^32^, http://jupyter.org/) used to generate the chart, and the underlying data required to reproduce such chart. In order to provide a means to graphically represent data provenance, the workflow can be interactively visualized on demand based on the graph information encoded in the metadata; each node (dataset, script, chart, measurement, simulation) can be further expanded with the possibility to be redirected on demand to the physical location of the raw data (measured and/or computed). In this way, the GUI of Qresp facilitates the reproducibility and repurposing of research data by integrating the paper with machine-readable digital content that describes experiments, apparatuses, raw materials, computational codes, calculation inputs, and outputs. The partial or full transfer of the large volume of data can optionally be leveraged by faster and secure gridFTP protocols, e.g. Globus^33,34^. An example of paper exploration is presented in Fig. 3.

## Discussion

The premises on which Qresp is built is that data associated to scientific publications are organized, curated and owned by the authors, with no requirements to be deposited in any central database and storage; rather they are simply made accessible and searchable through the use of distributed metadata collections. In short, the Qresp strategy is that of a distributed model of federated nodes; hence it is easily deployable by interested publishers, who may link to data exposed by the authors and/or mirror them, thus facilitating both the peer-review process and readers’ access. In addition, the organizer feature of Qresp allows the authors to version and track the history of changes to their data using Git (https://git-scm.com/).

We emphasize that the advantage of Qresp is not only to facilitate data reproducibility; through the creation of the paper workflow, Qresp enables authors to explain and make public all the technical parts of a paper that are usually relegated to the Supporting Information. Qresp does not lock researchers in the utilization of Qresp-enabled instruments/software and can be used to organize research data *a posteriori*, i.e. after the results are obtained and analyzed. However, the utilization of the Qresp organizer encourages the adoption of good scientific practices from the early stages of a research project, by encouraging the use (and re-use) of scripted procedures that reduce the number of time-consuming and error-prone manual operations. Qresp can be an important tool to foster a culture of data management and sharing.

Qresp also serves the need of facilitating data mining and statistical analysis/learning. Many groups are exploring various machine learning strategies to solve a variety of problems in science. Qresp will greatly increase the availability of a large amount of data to be mined for statistical learning, thus contributing to the expansion and robustness of the field.

Although some amount of irreproducibility of any scientific investigation is probably inevitable the strategy proposed here is expected to increase experimental and computational rigor in reporting results, together with transparency, and to greatly facilitate paper reproducibility. In addition, it will help decrease the time frame for sharing data and knowledge amongst investigators belonging to a certain community or research group, thus increasing productivity and minimizing duplication of efforts and costs. Finally, Qresp represents a social and technical infrastructure solution that will facilitate the organization of research data for reuse and repurpose, as mandated by federally funded research projects. A paper organized and curated with Qresp becomes an active and sharable piece of research work, where the information shared encompasses data and scientific procedures.

## Methods

The Qresp software is available at http://qresp.org/ with documentation and tutorials.

Qresp is Python software that uses the Flask framework (http://flask.pocoo.org) for the web application development. To ease installation and distribution of Qresp, we have used Docker containers (https://www.docker.com/) as specified in the Qresp documentation.

Steps and procedures used in producing the data curated and explored by Qresp are described by the workflow generated by Qresp (see Fig. 2). All data outputs are available through the explorer interface of Qresp. A running instance of Qresp is installed at https://paperstack.uchicago.edu/. Although Qresp does not require a specific organization of data, we used the structure suggested in Fig. 2 to organize the papers for curation, and have adopted the following data structure:

- **datasets**. This folder contains raw data generated in the scientific paper i.e. datasets created by an instrument or by a versioned software.
- **charts**. This folder contains the images (e.g. figure.png) of figures and tables and the notebooks (e.g. figure.ipynb) used to create them. Data files (e.g. figure.csv) contain exclusively the data displayed in the figures and tables.
- **scripts**. This folder contains source codes (e.g. script.py) not available publicly, used to manipulate **datasets** and generate the data files of **charts**, or other data discussed in the scientific paper.
- **tools**. This folder contains patches of publicly available or versioned software (e.g. modifications.diff), customized by the user to generate some of the **datasets**.
- **doc**. This folder contains documentation (e.g. readme.txt) that the user may want to provide in addition to that reported in notebooks and in the scientific paper, for example, tutorials.
- **toc.ipynb**. This notebook file serves as a table of contents and may contain links to all **datasets**, **charts**, **scripts**, **tools**, and **doc**.

All data organized by Qresp and described here must be located on servers accessible to the public and running an HTTP service. Although optional, both Globus (https://www.globus.org/) and Git (https://git-scm.com/) may be installed on such a server to deploy download via gridFTP and version control, respectively. Multiple instances of Qresp form a Qresp ecosystem, where each node of the network can be queried using a federated search mechanism. Qresp instances are made discoverable for example by a connection to a public registry (http://qresp.org/), that collects only the locations of running Qresp instances.

The metadata file generated by the Curator is encoded using the JSON (JavaScript Object Notation, http://json.org/) syntax. The version 1.0 of the JSON schema may be downloaded (http://qresp.org/Download.html).

When an instance of Qresp is created, the user needs to connect to an existing MongoDB (https://www.mongodb.com) database to collect the metadata. Establishing this connection is required only once: when Qresp is first installed. Qresp exposes REST APIs to manage the insertion of content into the database, as described in the Qresp documentation.

A permanent identifier can be assigned to the curated content. Digital Object Identifier (DOI) minting is interfaced with the Figshare (https://figshare.com) minting process, as described in detail in the documentation.

### Code availability

Version 1.0 of Qresp can be accessed via Zenodo (http://doi.org/10.5281/zenodo.2399865).

### Data availability

No new datasets were generated or analyzed during the current study.

## Additional information

**How to cite this article**: Govoni, M. *et al*. Qresp, a tool for curating, discovering and exploring reproducible scientific papers. *Sci. Data*. 6:190002 https://doi.org/10.1038/sdata.2019.2 (2019).

**Publisher’s note**: Springer Nature remains neutral with regard to jurisdictional claims in published maps and institutional affiliations.
